# Macrophage MicroRNAs as Therapeutic Targets for Atherosclerosis, Metabolic Syndrome, and Cancer

**DOI:** 10.3390/ijms19061756

**Published:** 2018-06-13

**Authors:** Yuanyuan Wei, Mengyu Zhu, Andreas Schober

**Affiliations:** 1 Experimental Vascular Medicine, Institute for Cardiovascular Prevention, Ludwig-Maximilians-University Munich, 80336 Munich, Germany; Yuanyuan.Wei@med.uni-muenchen.de (Y.W.); Mengyu.Zhu@med.uni-muenchen.de (M.Z.); 2 German Center for Cardiovascular Research (DZHK), Partner Site Munich Heart Alliance, 80802 Munich, Germany

**Keywords:** immune disorders, microRNA, macrophage, lipid metabolism, chronic inflammation

## Abstract

Macrophages play a crucial role in the innate immune system and contribute to a broad spectrum of pathologies in chronic inflammatory diseases. MicroRNAs (miRNAs) have been demonstrated to play important roles in macrophage functions by regulating macrophage polarization, lipid metabolism and so on. Thus, miRNAs represent promising diagnostic and therapeutic targets in immune disorders. In this review, we will summarize the role of miRNAs in atherosclerosis, metabolic syndrome, and cancer by modulating macrophage phenotypes, which has been supported by in vivo evidence.

## 1. Introduction

The innate immune system is central for the maintenance of tissue homeostasis and quickly responds to physiological and pathological stimuli such as tissue injury and infection. In response to tissue injury, a multifactorial network of chemical signals initiates and maintains a host response designed to ‘heal’ the afflicted tissue. Innate immune cells recognize general patterns associated with pathogen infection and damaged cells, and perform nonspecific elimination of the pathogen either by cellular mechanisms such as macrophages or natural killer (NK) cells, or humoral mechanisms such as the complement system. Innate immune cells (in particular, macrophage)-mediated low-grade chronic inflammation plays a central role in pathological process of several diseases, such as atherosclerosis, obesity-induced metabolic syndrome, and cancer. The role of miRNAs in macrophages has been summarized somewhere else [1,2], here we will review macrophage-derived miRNAs that have recently come to light as affecting atherogenesis, obesity-induced metabolic syndrome and cancer, which is supported by in vivo evidences.

### 1.1. Macrophage Plasticity and Polarization

Macrophages belong to the mononuclear phagocyte system (MPS), which is defined by the origin from bone marrow-derived cells and by the capability of phagocytosis, cytokine secretion and antigen presentation [3,4]. Cells of the MPS have a great capacity to specialize in particular during an inflammatory response where monocytes are recruited into the tissues and differentiate into macrophages. In response to environmental cues, macrophages can polarize mainly into two distinct functional phenotypes [5,6]. Lipopolysaccharides (LPS) in combination with the Th1 cytokine interferon gamma (IFN-γ) induce a proinflammatory macrophage phenotype (also termed classically activated or M1-type macrophages), which is characterized by nuclear factor (NF-κB)- and signal transducer and activator of transcription 1 (STAT1)-dependent upregulation of proinflammatory genes, such as interleukin (IL)-1β and tumor necrosis factors (TNF)-α [3,7,8]. By contrast, stimulation with IL-4 promotes an anti-inflammatory macrophage phenotype (termed alternatively activated or M2-type macrophages), characterized by the expression of mannose receptor, C type 1 (Mrc1), Retnla resistin like alpha (Retnla, also known as Fizz1), or chitinase-like 3 (Chil3, also known as Ym1) in mouse [9,10,11]. Moreover, M1- and M2-type macrophages differentially utilize l-arginine. Whereas IFN-γ upregulates nitric oxide synthase 2 (NOS2) to generate nitric oxide (NO) from arginine, IL-4 promotes arginase-dependent formation of l-ornithine [12]. Although the classification of these two distinct macrophage populations is useful for experimental purposes, it is important to appreciate that it is an oversimplified in vitro model. In vivo, macrophages are a heterogeneous population and can display phenotypes across the spectrum from anti- to pro-inflammatory.

Similar to rapidly dividing cells and in contrast to most differentiated cell types, macrophages convert glucose into lactate at a high rate in the presence of oxygen (termed aerobic glycolysis or Warburg effect) while very little of the glucose is oxidized [13,14]. In addition, utilization of long-chain fatty acids for mitochondrial oxidative phosphorylation (OXPHOS) plays an important role in the energy generation in macrophages [13,15]. Polarization of resting macrophages alters their mode of ATP generation [16]. Aerobic glycolysis is enhanced in inflammatory macrophages by the activation of hypoxia-inducible factor-1 and is essential for ATP production in these cells, whereas fatty acid oxidation (FAO, also termed β-oxidation) is primarily utilized to produce mitochondrial reactive oxygen species (ROS), which is important for bactericidal activity [16,17,18]. The low rate of ATP production by OXPHOS due to the inhibitory effect of NO on cytochrome c oxidase may contribute to the increased ROS production in inflammatory macrophages [19,20,21]. By contrast, glucose consumption is much lower in M2- compared to M1-macrophages, whereas upregulation of ATP synthesis by oxidative metabolism of fatty acids promotes the anti-inflammatory phenotype and improves the survival of M2-macrophages [18,22], indicating that differences in nutrient utilization control macrophage activation. M2-polarization by IL-4 is mediated through the upregulation of the transcriptional coactivator peroxisome proliferator-activated receptor coactivator-1 beta (PGC-1β), which increases the expression of genes related to oxidative metabolism [22]. Accordingly, the transition from early to late inflammatory response is associated with a metabolic switch in macrophages from increased glycolysis to increased FAO [23].

### 1.2. MicroRNA

MicroRNAs (miRNAs) are small (about 20–22 nt), non-coding RNAs, which negatively regulate gene expression by translational inhibition or mRNA decay. The RNase Dicer cleaves the hairpin miRNA precursors into miRNA duplexes [24]. One strand of the miRNA duplex is usually incorporated into the miRNA-induced silencing complex (mRISC) through the Argonaut (Ago) proteins, such as Ago2, which guides the binding of the nucleotides 2–8 of the 5′ end of miRNAs to a complementary sequence in the 3′UTR of the target mRNAs. Because of the short seed sequence and its often imperfect complementary binding to the mRNA recognition element, an individual miRNA can affect the expression of hundreds of mRNAs [25]. In addition, miRNAs have been found to be secreted to the extracellular space by encapsulated in extracellular vesicles (EVs), such as exosomes and apoptotic bodies, which can be taken up by neighboring or distant recipient cells [26]. miRNAs can also be found inside high-density lipoproteins (HDLs), or bound by Ago2 outside of vesicles [27,28,29].

## 2. Atherosclerosis

### 2.1. Macrophages in Atherosclerosis

Atherosclerosis is a leading—but potentially preventable—cause of death and disability worldwide resulting in devastating diseases, such as myocardial infarction due to coronary artery disease or stroke [30]. The current concept of atherogenesis includes a central role of apolipoprotein B-containing lipoproteins, such as low-density lipoproteins (LDL) or remnant lipoproteins, which trigger a chronic inflammatory response of the vessel wall dominated by the infiltration of monocyte-derived macrophages. Reduced shear stress and increased permeability for lipoproteins due to endothelial cell turnover is responsible for subendothelial retention of LDL in atherosclerosis-prone regions of medium-sized arteries, such as branching points or curvatures. Modifications of LDL, like oxidation, in the vessel wall induce the expression of adhesion molecules (e.g., vascular cell adhesion molecule-1) and chemokines in the endothelium, thus supporting the adhesion and transmigration of circulating monocytes. In the vessel wall, monocytes differentiate into macrophages, which transform into “foam cells” by intracellular accumulation of cholesterol [31]. Progression of atherosclerotic plaques is driven by the inflammatory activation of lipid-laden macrophages due to an increase of free cholesterol and the formation of cholesterol crystals [32,33]. Ultimately, elevated free cholesterol triggers endoplasmic reticulum (ER) stress, which causes macrophage apoptosis [33]. In early atherosclerosis, macrophage apoptosis reduces lesion formation by reducing the lesional macrophage content and the anti-inflammatory effects of efferocytosis. By contrast, phagocytosis of apoptotic macrophages is impaired in advanced atherosclerosis resulting in secondary necrosis and subsequent release of proinflammatory factors, which fuels the formation of a highly thrombogenic necrotic core [34].

Multiple stimuli, including cytokines, chemokines and modified lipoproteins, may dynamically modulate the macrophage phenotype in atherosclerotic lesions. In general, inflammatory macrophages promote atherosclerosis progression, whereas M2-like macrophages carry out processes that can suppress plaque progression [35].

### 2.2. miRNAs Regulate Foam Cell Formation in Atherosclerosis

**Figure 1:**
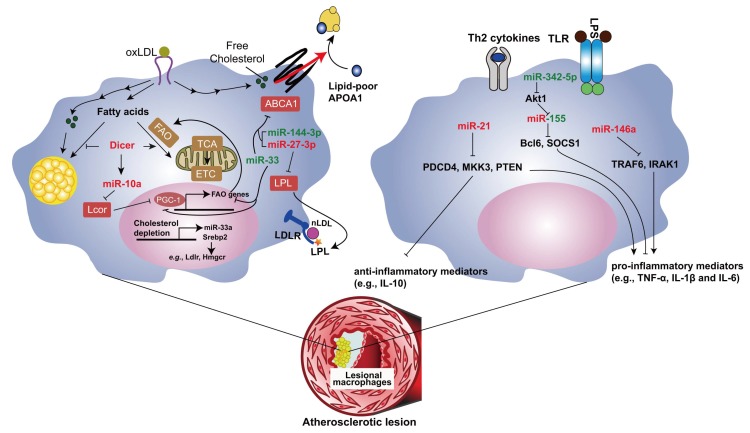
Role of miRNAs in atherosclerosis by regulating lesional macrophage phenotypes. Dicer plays an athero-protective role by enhancing fatty acid oxidation (FAO) in foam cells through generating miR-10a. miR-33 and miR-144-3p promote foam cell formation by inhibiting ABCA1, thereby foster atherosclerosis. Although miR-27-3p targets ABCA1, it limits atherosclerosis through inhibiting LPL-mediated cholesterol uptake. miR-155 enhances advanced atherosclerosis by promoting inflammatory macrophage activation, whereas miR-21 and miR-146a limits atherosclerosis by inhibiting inflammatory macrophage activation. However, at the early stage, miR-155 limits atherosclerosis by repressing macrophage proliferation. miR-342-5p promotes atherosclerosis by upregulating miR-155 and enhancing macrophage inflammation. The red color indicates the athero-protective miRNAs, whereas the green color indicates the atherogenic miRNAs. The black arrow indicates the promoting effect, and the red arrow indicates the reverse cholesterol transport. The T bar indicates the inhibitory effect.

miRNAs play a central role in foam cell formation by regulating several steps involved in cholesterol and fatty acid metabolism. Our study shows that mitochondrial FAO is enhanced by oxidized LDL during foam cell formation, which protects macrophages from lipid overloading [36]. Impaired miRNA processing by deletion of *Dicer* gene enhances lipid accumulation due to repressed FAO in murine macrophages (Figure 1). Thus, Dicer in macrophages reduces the lipid burden in the plaque and limits the progression of atherosclerosis in the mouse model [36]. This effect of Dicer is mediated by generating miRNAs, such as miR-10a, let-7b and miR-195a. Among those miRNAs, miR-10a limits foam cell formation by promoting FAO in macrophages and reduces atherosclerosis through targeting ligand dependent nuclear receptor corepressor (Lcor). Although our study demonstrates that let-7b and miR-195a positively regulate FAO in macrophages, their targets involved need to be further investigated. Therefore, promoting Dicer/miR-10a-mediated FAO might be a novel potential therapeutic strategy of atherosclerosis.

Given the individual miRNAs, the role of the highly conserved miRNA miR-33 in lipid metabolism has been extensively studied (Figure 1). Human miR-33 miRNA family consists of miR-33a and miR-33b, which are encoded by an intron within sterol regulatory element binding transcription factor (*SREBP*)*-2* and *SREBP-1* gene, respectively [37]. However, only miR-33a homolog was found in mice (referred to here as miR-33). SREBP-2 is a key transcription factor in cholesterol metabolism by inducing expression of the LDL receptor and cholesterol biosynthesis genes, whereas SREBP-1 primarily promotes fatty acid synthesis. In both mouse and human, miR-33 targets the 3′UTR of several genes involved in cholesterol homeostasis including ATP binding cassette subfamily A member 1 (ABCA1) and thereby limit cholesterol efflux from macrophages to ApoA1 and increase macrophage apoptosis induced by free-cholesterol loading [38,39,40]. In mouse, but not human macrophages, miR-33 also targets ATP binding cassette subfamily G member 1 (ABCG1), thus inhibiting cholesterol efflux to HDL [38]. Moreover, miR-33 targets PGC-1α in both human and mouse macrophages, thereby inhibiting mitochondrial ATP production required for the ATP-dependent cholesterol efflux via ABCA1 [41]. In addition to PGC-1α [42,43,44], miR-33 targets several genes involved in FAO, such as carnitine palmitoyltransferase 1A, hydroxyacyl-CoA dehydrogenase, Sirtuin 6, and AMP kinase subunit-α [45,46]. Thus, miR-33 may promote foam cell formation by inhibiting FAO.

miR-33a expression was increased in the plasma from atherosclerotic patients, and miR-33a/b was upregulated in human carotid atherosclerotic plaques compared with normal arteries [41,47]. Macrophage-specific *miR-33* deficiency reduces lipid accumulation and inflammation, resulting in reduced atherosclerotic plaque burden in hyperlipidemic mice [48]. Similarly, systemic inhibition of miR-33 reduces atherosclerosis progression [49,50].

Several other miRNAs also inhibit reverse cholesterol transport through targeting ABCA1, such as miR-144-3p [51] (Figure 1). miR-144-3p inhibits cholesterol efflux, whereas enhances secretion of inflammatory mediators, including TNF-α, IL-1β and IL-6, from foam cells both in vitro and in vivo. Expression level of this miRNA was upregulated in patients with acute myocardial infarction. Treatment with the miR-144-3p mimic promotes the progression of atherosclerosis in *Apoe* deficient mice [51].

In addition to target ABCA1, miR-27a/b-3p limits the uptake of cholesterol partly by targeting lipoprotein lipase (LPL) that retains atherogenic lipoproteins through forming a nonenzymatic bridge between lipoprotein receptors and proteoglycans in subendothelial spaces [52,53]. Moreover, miR-27a/b-3p repress the production of inflammatory mediators, such as IL-1β, IL-6, monocyte chemotactic protein 1 (MCP1, also known as CCL2) and TNF-α, in foam cells. Forced overexpression of miR-27a/b-3p inhibits, whereas inhibition of miR-27a/b-3p promotes the development of atherosclerosis in *Apoe* deficient mice [54]. These data indicate that the inhibitory effect of miR-27a/b-3p on cholesterol uptake is more prominent than that on cholesterol efflux, which leads to its atheroprotective net effect.

### 2.3. miRNAs Regulate Macrophage Inflammation in Atherosclerosis

The role of miRNAs in macrophage inflammation during atherosclerosis has been studied intensively. Both pro- and anti-inflammatory functions of miRNAs have been reported. Impaired miRNA process by *Dicer* deletion reprogrammed alternatively activated macrophages towards a proinflammatory feature in vitro and upregulates the expression of proinflammatory mediators in mouse atherosclerotic lesions [36].

miR-21 is the most abundant miRNA in macrophages, and overexpression of miR-21 promotes IL-10 and represses IL-1β expression in macrophages [55,56,57], indicating the anti-inflammatory role of miR-21. miR-21 was significantly downregulated in both circulation and unstable atherosclerotic lesions from patients with advanced carotid artery disease [58,59]. Either global or bone marrow-specific deletion of *miR-21* accelerates atherosclerosis, promotes vascular inflammation and enhances foam cell formation in hyperlipidemic mice [59,60]. Of note, local delivery of a miR-21 mimic using ultrasound-targeted microbubbles into carotid plaques prevents advanced plaques from rupture in mice [59]. The anti-atherosclerotic role of miR-21 might be the integral effect of its anti-inflammatory role and inhibitory role in lipid uptake by targeting several genes, such as programmed cell death 4 (PDCD4), mitogen-activated protein kinase kinase 3 (Map2k3, also known as MKK3) and phosphatase and tensin homolog (PTEN) [56,60,61]. Because miR-21 is a notorious tumorigenic miRNA [62,63], local delivery rather than systemic treatment might be the appropriate therapeutic administration of miR-21 in atherosclerosis.

miR-155 is the most studied proinflammatory miRNA in atherosclerosis. This miRNA is crucial for the classical activation of macrophages and promotes the expression of inflammatory mediators, such as TNF-α, MCP1 and IL-6 by targeting B cell leukemia/lymphoma 6 (Bcl6) and suppressor of cytokine signaling 1 (SOCS1) [64,65,66,67]. Expression of miR-155 is increased in both murine and human lesions with the development of atherosclerosis [64,65,68]. Both pro- and anti-atherosclerotic role of miR-155 have been reported [64,65,68,69]. These controversial findings may be due to a stage-dependent effect of miR-155 [68], because of the dynamic changes of lesional macrophage subtypes and miR-155 targets during the progression of atherosclerosis. In mice, miR-155 represses colony stimulating factor 1 receptor (Csf1r) expression, reduces lesional macrophage content and proliferation and decreases lesion size at the early stage of atherosclerosis [68]. However, in advanced lesions, the role of miR-155-regulated Csf1r expression in lesional macrophage accumulation is limited owing to the downregulation of macrophage colony-stimulating factor (M-CSF). On the other hand, targeting of Bcl6 by miR-155 fosters atherosclerosis due to increased MCP1 level and impaired macrophage efferocytosis at the late stage in mice [65,68]. The stage-dependent role of miR-155 suggests that blocking the interaction between miR-155 and Bcl6 might be a potential therapeutic strategy for atherosclerosis.

The function of another miRNA, miR-342-5p, is closely connected to miR-155 in macrophages. miR-342-5p promotes inflammatory activation of murine macrophages by targeting a negative regulator of miR-155, thymoma viral proto-oncogene 1 (Akt1) in M1 macrophages [66]. In resting macrophages, the endogenous competitive RNA bone morphogenetic protein receptor type-2 (Bmpr2) prevents targeting of Akt1 by miR-342-5p. However, the transcriptional suppression of Bmpr2 during M1 polarization increases the availability of miR-342-5p and thereby leads to increased targeting of Akt1 by miR-342-5p. Therefore, the competition between Bmpr2 and Akt1 for the binding to miR-342-5p regulates the expression of miR-155. miR-342-5p is mainly expressed in lesional macrophages and upregulated during the progression of atherosclerosis in mice. Inhibition of miR-342-5p either locally or systemically limits the development of atherosclerosis in *Apoe* deficient mice [66]. Thus, miR-342-5p and miR-155 act as a functional pair in the proatherogenic activation of macrophages.

LPS-induced toll-like receptor activation and proinflammatory cytokines upregulate miR-146a expression via proinflammatory NF-κB signaling, resulting in endotoxin tolerance and limiting IL-1β-induced inflammatory activation by repressing TNF receptor-associated factor 6 (TRAF6) and IL-1 receptor-associated kinase 1 (IRAK1) [70,71,72]. In hyperlipidemic mice, treatment with miR-146a mimics reduces Ly-6C^high^ monocytosis, the lesional macrophage number, the macrophage inflammatory response, and atherosclerosis [73].

Regulation of lipid metabolism and inflammatory activation in macrophages are both crucial in the development of atherosclerosis, however, only one aspect was studied in most researches on miRNA functions in macrophages. Thus, finding the miRNAs that could integrate both aspects of macrophages is required to develop the new therapeutic strategy of atherosclerosis.

## 3. Obesity-Induced Metabolic Syndrome

### 3.1. Macrophage, Inflammation and Obesity

Obesity, characterized by excess accumulation of adipose tissue, has become epidemic proportions worldwide. Obesity increases the risk of a number of diseases, including but not limited to type 2 diabetes, cardiovascular diseases and cancer [74,75]. Although once obesity was considered to be a simple lipid-storage disease, the notion that obesity is a chronic inflammatory process has now been accepted broadly [76]. Obesity-associated metabolic inflammation is the key cause of insulin resistance and impaired glucose metabolism [76]. The pathophysiological link between obesity, inflammation, and insulin resistance was first demonstrated when the researchers found the secretion of TNF-α from the adipose tissue of obese rodents [77]. Accumulation of macrophages in adipose tissue is the principle source of inflammatory mediators, including IL-6, IL-1β, MCP1, and macrophage inhibitory factor (MIF) in addition to TNF-α [78,79,80]. Depending on the local microenvironment, adipose tissue macrophages (ATMs) can span the spectrum from an anti-inflammatory to a proinflammatory phenotype. The resident macrophages, such as those present in adipose tissue of lean mice, display the alternatively activated phenotype and support adipose homeostasis [81]. During obesity, inflammatory mediators released from adipose tissue, such as saturated fatty acids, cytokines, and IFN-γ, induce an increase in the recruitment of M1-like macrophages [82]. Moreover, ATMs show phenotypic plasticity and can modify their phenotypes in response to changes in the local microenvironment [83].

### 3.2. miRNAs Control Obesity-Related Immunometabolic Diseases by Regulating Macrophage Polarization

**Figure 2:**
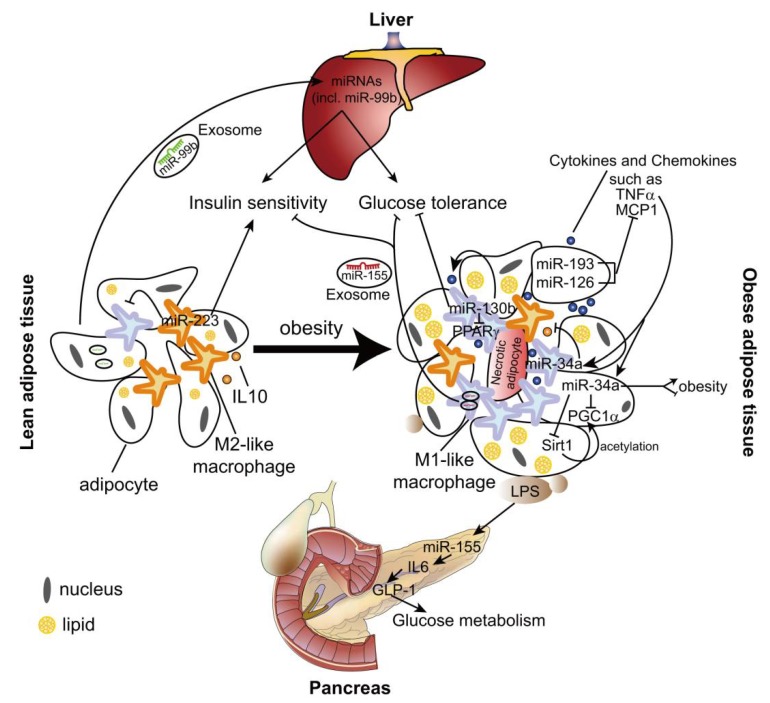
Role of miRNAs in obesity-induced metabolic syndrome by regulating macrophage phenotypes. Obesity upregulates the expression of several miRNAs, like miR-34a and miR-130b, which promote the inflammatory activation of macrophages. Obesity also changes the miRNA profiles in ATM-derived exosomes, which mediates the communication between adipose tissues and the other organs. Upregulation of miR-155 in ATM-Exos induces insulin resistance and glucose intolerance in the liver. Induction of miR-155 expression by hyperlipidemia-associated endotoxemia promotes *IL-6* gene transcription and increases production of Glucagon-like peptide-1 (GLP-1) in pancreas, which promotes glucose metabolism. Moreover, in the lean mice, miR-223 inhibits the inflammatory activation of ATMs and promotes insulin sensitivity. miRNAs, such as miR-99b, packaged in adipocyte-derived exosomes increase insulin sensitivity and glucose tolerance by regulating gene expression in liver. The arrow indicates the promoting effect and the T bar indicates the inhibitory effect.

Like atherosclerosis, several miRNAs may contribute to the pathological process of obesity-related immunometabolic diseases by regulating macrophage polarization. miR-193 and miR-126 inhibit MCP1 expression in both adipocytes and macrophages (Figure 2), which may suppress M1 polarization of ATMs [84]. These two miRNAs are downregulated in adipose tissue and adipocytes from obese men [84], although it is not known whether their expression in ATMs is changed by obesity. However, the role of miR-193 and miR-126 in obesity-induced metabolic syndrome needs to be validated in the animal models.

miR-223 is a hematopoietic miRNA and mainly expressed in ATMs, but barely detected in adipocytes [85]. IL-4 stimulation induces miR-223 expression in murine macrophages in a peroxisome proliferator activated receptor gamma (PPARγ)-dependent manner [85,86], whereas LPS treatment downregulates the expression level of miR-223 [85,87]. Deletion of *miR-223* leads to more M1-like ATMs, which results in enhanced adipose tissue inflammation and systemic insulin resistance in the diet-induced obese mouse model [85]. Although Pbx/knotted 1 homeobox (Pknox1, also known as Prep-1) was identified as a direct target of miR-223 and mediates the anti-inflammatory effect of miR-223, it is not known if Pknox1 is responsible for the effect of miR-223 on ATM polarization in vivo [85].

miR-34a is expressed in both adipocytes and macrophages [88]. TNF-α upregulates the expression of miR-34a in murine bone marrow-derived macrophages (Figure 2). Deletion of *miR-34a* in high fat diet (HFD)-fed mice causes more ATMs exhibiting F4/80^high^ phenotype, which is characterized by high levels of anti-inflammatory IL-10 [88,89]. However, there is no difference in the expression of the other M1/M2 markers in *miR-34a* deficient ATMs. Moreover, *miR-34a* deficiency promotes the expression and Sirt1-mediated acetylation of PGC-1α, which may enhance fatty acid oxidation and lipolysis in the obese adipose tissue [88,90]. Surprisingly, HFD-fed *miR-34a* KO mice were significantly heavier with a greater increase in white adipose tissue weight than WT [88]. However, lentiviral-mediated inhibition of miR-34a reduces the body weight of obese mice [90]. The opposite effects of miR-34a might be the result from different mouse models used in these two studies: the uptake of lentivirus-vector might differ in various cell types, which may cause the different knockdown efficiency of miR-34a between ATMs and adipocytes in [90], whereas *miR-34a* is globally deleted in [88]. Thus, the cell-specific effect of miR-34a on obesity and metabolic syndrome should be further studied in the future.

MiR-130b is upregulated in macrophages of HFD-fed mice, which promotes M1 macrophage polarization via targeting PPARγ. Transplantation of macrophages pretreated with miR-130b inhibitor attenuates adipose tissue inflammation and glucose intolerance in mice fed on an HFD [91].

### 3.3. miRNAs Secreted from Adipose Tissue Influence the Distant Tissues

The extensive communications between adipose tissue and other insulin target tissues, pancreatic islets or cardiovascular system result in the causal link between obesity and other immunometabolic disorders, such as diabetes and cardiovascular diseases. There are many substances released from adipose tissue that travel through the circulation to influence distant organs, including cytokines, lipid species, adipokines, and RNA-containing microvesicles [92].

Microvesicles are small (30–1000 nm) membrane-covered vesicles derived from a couple of cell types, including both macrophages and adipocytes from the adipose tissues [93,94,95,96]. Microvesicles include several extracellular organelles, such as exosomes, shedding microvesicles and apoptotic bodies, many of which exhibit pleiotropic biological functions [96]. One of the most important roles of these microvesicles is working as the carrier of RNA molecules to mediate intercellular communication. Approximately 7000 mRNAs and 140 miRNAs are present in adipocyte-derived microvesicles (ADMs), and about 500 miRNAs are identified in ATM-derived exosomes (ATM-Exos) [97,98]. The presence of endogenous Ago2 in adipose tissue-derived microvesicles indicates the functional role of miRNAs in such kind of microvesicles. For example, ADM miRNAs increase insulin sensitivity and improve glucose tolerance through regulating liver gene expression by the transferred miRNAs [99,100]. Moreover, obesity induces changes of miRNA expression in ATM-Exos, suggesting the pathological role of these miRNAs in obesity [98].

In contrast to lean mice, obesity causes upregulation of miR-155 expression in both ADMs and ATM-Exos. By targeting PPARγ, miR-155 mediates the inhibitory effect of ATM-Exos from obese mice on the cellular insulin sensitivity in liver and muscle [98]. Moreover, uptake of miR-155 derived from ADMs represses SOCS1 expression in macrophages, which leads to enhanced expression of proinflammatory mediators, such as TNF-α and iNOs, whereas reduced expression of M2 markers, Arginase-1 and Ym1 [101]. These studies indicate the pathological role of miR-155 in obesity-induced chronic inflammation and metabolic syndrome.

In addition to regulate macrophage phenotypes, miR-155 mediates adipocyte dysfunction caused by inflammatory cytokines (e.g., TNF-α), which may contribute to the diet-induced obesity progression in C57BL/6 mice by limiting brown adipose tissue differentiation [102,103,104]. By contrast, in hyperlipidemic *Ldlr^−/−^* and *Apoe^−/−^* mice, *miR-155* deficiency aggravates obesity, which is accompanied by augmented gonadal adipocyte hypertrophy and macrophage recruitment in adipose tissues [105,106]. This effect may be mediated by the beneficial role of miR-155 in glucose homeostasis during hyperlipidemia [105]. Hyperlipidemia-associated endotoxemia leads to an increase in miR-155 expression in pancreatic islets in lean mice, which improves glucose metabolism by promoting IL-6-induced production of Glucagon-like peptide-1 (GLP-1) and pancreatic β-cell adaptation. In *Ldlr^−/−^* mice fed on an HFD, elevated plasma GLP-1 levels by miR-155 may limit the progression of obesity and adipose tissue inflammation [105]. Accordingly, global transgenic overexpression of miR-155 improves glucose tolerance and insulin sensitivity by inhibiting the negative regulators (e.g., C/EBPβ, HDAC4 and SOCS1) of insulin signaling in lean mice [107]. The variable roles of miR-155 in different cell types might be the reason for the controversial effects of miR-155 on obesity, which can be studied by using cell-specific *miR-155* knockout mouse model.

These studies indicate that miRNAs present in ADMs and ATM-Exos play an opposite role in regulating glucose tolerance and insulin sensitivity, which might be the result from different kinds of miRNAs packaged in the exosomes. During obesity, the metabolic organs may uptake the exosomes derived from both cell types in the adipose tissue, however, the net-effect of the exosomal miRNAs from adipose tissue is still unknown. Therefore, further investigate the miRNA profiles in both ADMs and ATM-Exos and the effect of individual miRNAs on the distant organs will be necessary in the future.

## 4. Cancer

A possible link between inflammation and cancer is first indicated by the presence of leukocytes in neoplastic tissues, observed by Rudolf Virchow in 1863 [108]. During the last decade, clearer evidence supports the crucial role of inflammation in tumor growth, progression, and immunosuppression. About 15–20% of cancers, such as cervical carcinoma and gastric cancer, are attributable to chronic bacterial and viral infections, and more than half of cancers are associated with chronic inflammation induced by environmental factors, such as tobacco smoking and dietary factors [109,110]. Conversely, in other types of cancers, an oncogenic change triggers an inflammatory response that promotes tumorigenesis [111]. Based on the link between cancer and inflammation, targeting the inflammatory components of the tumor microenvironment could promote the development of a new therapeutic strategy for prevention and treatment of cancers.

### 4.1. Tumor-Associated Macrophages

The inflammatory microenvironment of tumors contains innate immune cells (for example, macrophages, neutrophils and dendritic cells) and adaptive immune cells (T and B lymphocytes) both in tumor areas and the supporting stroma [112,113]. Tumor-associated macrophages (TAMs), derived from circulating monocytes that are recruited into the tumor sites by MCP1 chemokines, are the major component of infiltrating leucocytes in neoplastic tissues. Although most mouse studies support a crucial role of TAMs in malignant progression by depletion of macrophages in a couple of mouse tumor models [114,115,116,117], clinical studies implicate both pro- and anti-tumoral functions of TAMs in different cancers [118,119,120,121,122,123,124]. The discrepancy between studies may be the result from different macrophage subsets existing in the tumor microenvironment. A majority of TAMs is described as an M2-like population that enhances tumor progression by supporting angiogenesis, promoting tumor cell invasion and metastasis, and suppressing adaptive immunity [117,125]. However, some studies suggest the association of better prognosis with M1-like population that are potent effector cells killing tumor cells and produce proinflammatory cytokines [122,126,127]. Additionally, TAMs are composed of several distinct populations overlapping inflammatory and immunosuppressive features as a result of complex instructions given by the tumor microenvironment [128,129,130,131]. Moreover, a temporal plasticity of TAMs is indicated by the fact that macrophages show an inflammatory phenotype in the early phase of tumor establishment, while displaying a pro-tumoral or immunosuppressive phenotype in the later phase of tumor progression [132]. Therefore, repolarizing TAMs to immunostimulatory phenotypes might be a potential therapeutic strategy of cancer.

Of note, some differences have been reported between mouse and human macrophages. For example, human macrophages are different from mouse macrophages in their nitric oxide (NO) production. In mouse macrophages, inducible NO synthase (iNOS), induced by LPS and IFN-γ, is responsible to produce large amount of NO [133]. However, despite the expression of iNOS mRNA and protein, the abilities of iNOS to generate NO in human macrophages are very low [134,135]. Moreover, Arginase-1 and Ym1 are markers for mouse, but not human, M2 macrophages [136]. These discrepancies between humans and mice might reflect our incomplete understanding of macrophage phenotypes involved in cancer, which may cause the controversy as to the role of TAMs.

### 4.2. miRNAs Control Tumorigenesis by Regulating Inflammatory Macrophage Activation

miRNAs play a key role in regulating TAM phenotypes in cancer (Table 1). Generation of miRNAs by Dicer prompts M2-like TAM polarization and inhibits tumor infiltration of CD8^+^ cytotoxic T lymphocytes (CTLs) that enhances M1-like macrophage programming by producing IFN-γ, thus sustaining the immunosuppressive capacity of TAMs. At the molecular level, Dicer inhibits activation of type-I and -II IFN, signal STAT1, and IFN-regulatory factor (IRF) signaling, whereas positively regulates transforming growth factor-β (TGF-β), IL-10 and STAT6 signaling. Dicer promotes tumor development in the mouse model of orthotopic MMTV-PyMT mammary adenocarcinomas, subcutaneous LLC and subcutaneous MC38 carcinomas. Let-7d-5p partly contributes to the effects of Dicer on M2-like TAM phenotype and decreases tumor-infiltrating CTLs, however, has no effect on tumor growth. Therefore, which individual miRNAs are responsible for the pro-tumoral function of Dicer remains unknown [137].

miRNA expression profile is changed in tumor-associated myeloid cells in mice with invasive pancreatic ductal adenocarcinoma, for example, upregulation of miR-21-3p and -5p [131]. miR-21-3p and -5p probably inhibit migration of CTLs into tumor sites by suppressing CCL-3 and CXCL-10, respectively [131]. However, the in vivo evidence is lacking. miR-511-3p is upregulated in MRC1^+^ TAMs and suppresses tumor-promoting genes, including proteolytic enzymes and other extracellular matrix-remodeling molecules, thereby inhibiting tumor growth by targeting Rho-dependent kinase-2 (Rock2), a serine/threonine kinase that regulates cell cytoskeleton contractility [138,139]. In vitro evidences show that let-7b expression is upregulated in prostatic TAMs and promotes prostate carcinoma (PCa) cell mobility and angiogenesis [140].

In contrast, miR-155 expression is low in MRC1^+^ TAMs and inhibits tumor growth in a breast cancer mouse model by reprograming M2-like macrophages toward classic M1-like activation [142]. Although several genes have been identified as miR-155 targets, such as Ship1, bcl6 and PU.1 [65,150,151], it is still not clear which target mediates the anti-tumoral effect of miR-155. miR-142-3p is downregulated in glioblastoma-infiltrating macrophages, which contribute to the glioma growth probably by promoting M2-like TAM apoptosis through targeting transforming growth factor beta receptor 1 (TGFBR1) and transforming growth factor beta 2 (TGFB2) [143]. The expression of miR-19a-3p is decreased in TAMs in vitro and inhibits metastasis of 4T1 breast cancer cells by suppressing M2 macrophage function *i*n the mouse model, probably by upregulating Fra-1 expression [144].

### 4.3. Crosstalk between TAMs and Tumor Cells Mediated by miRNAs

Epithelial ovarian cancer (EOC)-derived exosomes promote M2-like phenotype of TAMs and accelerate progression of EOC by transferring miR-222-3p, which downregulates the expression of SOCS3 and activate STAT3 signaling pathway [146]. Colorectal cancer cells positively secreted miR-145 via EVs, which promotes M2-like polarization through the downregulation of histone deacetylase 11 [149]. Macrophages, that have taken up colorectal cancer cell-derived EVs, cause significant enlargement of the tumor size [149]. miR-940 expression is upregulated in the exosomes derived from epithelial ovarian cancer cells treated by hypoxia and in exosomes isolated from ascites of patients harbouring the epithelial ovarian cancer compared with the patients having benign ovarian diseases [147]. Macrophages taking up miR-940-involving exosomes exhibit M2 phenotypes, which promotes epithelial ovarian cancer cell proliferation and migration [147]. Furthermore, extracellular vesicles derived from hypoxic lung cancers activate M2 polarization by miR-103a transfer that inhibits PTEN expression [148]. However, the role of miR-940 and miR-103 on tumor growth in vivo is still unknown. 

Conversely, miR-21 is transferred from M2-macrophage to gastric cancer cells through exosomes and inhibits apoptosis of gastric cancer cells by regulating PTEN/phosphoinositide-3-kinase regulatory subunit 1 (Pik3r1, also known as PI3K)/Akt signaling and anti-apoptotic Bcl2 expression [152]. The exosomes derived from M2-macrophages accelerates the tumor growth [152]. 

### 4.4. Therapeutic Application of TAM-Derived miRNAs in Cancers

Cationic Bletilla Striata polysaccharide (cBSP), a polysaccharide isolated from an herb Bletilla striata and modified with *N*,*N*′-carbonyldiimidazole/ethylenediamine exhibits significantly high affinity to macrophages due to the expression of mannose receptor (MR) on the surface of macrophages, can be used as a nonviral nucleotide drug delivery system specifically targeting macrophages [153]. The nanocomplex packaging let-7b into cBSP can specifically release the cBSP & let-7b in response to the tumor acidic microenvironment in the aid of a pH-responsive material PEG-histamine-modified alginate (PHA) [141,154]. This multi-component complex efficiently reprograms TAMs towards M1-like and inhibits tumor growth in a breast cancer mouse model [141]. In another study, Teng et al. packaged miR-18a in a grapefruit-derived nanovector, which can be taken up by Kupffer cells, but not by hepatocytes after a tail vein injection [145]. miR-18a inhibits liver metastasis of colon cancer by inducing M1-like polarization due to increased IFN-γ production by targeting interferon regulatory factor 2 (IRF2). These data indicate the therapeutic application of macrophage-derived miRNAs by using macrophage-specific targeting systems.

## 5. Conclusions

miRNAs play crucial roles in many aspects of macrophages and thereby affect several immune disorders, like atherosclerosis, obesity-induced metabolic syndrome and cancer. Targeting miRNAs through the application of modified oligonucleotides may become an effective therapeutic strategy for immune diseases. However, the in vivo studies on miRNA functions are still limited. Moreover, the pathological functions of miRNAs are highly context- and cell type-dependent, like the controversial effect of miR-155 on obesity-induced diabetes. Unfortunately, the miRNA-regulated context-specific network is still poorly understood, which hampers the drug development. Therefore, further studies of miRNA functions in specific cell types in the in vivo disease models are required in the future.

## Figures and Tables

**Table 1:** The list of miRNAs controlling tumor growth by regulating macrophage phenotypes.

miRNA	Effect on TAMs	Effect on Tumor Growth	Potential Targets	Reference
let-7d	M2↑	→	CD86	[137]
let-7b	M1↑M2↓	↓	?	[141]
miR-511-3p	?	↓	Rock2	[138]
miR-155	M1↑M2↓	↓	?	[142]
miR-142-3p	apoptosis↑	↓	TGFBR1, TGFB2	[143]
miR-19a-3p	M1↑M2↓	↓	Fra-1	[144]
miR-18a	M1↑M2↓	↓	IRF2	[145]
miR-222-3p *	M2↑	↑	SOCS3	[146]
miR-940 *	M2↑	?	?	[147]
miR-103a *	M2↑	?	PTEN	[148]
miR-145 *	M2↑	↑	Histone deacetylase 11	[149]

* The miRNAs packaged in tumor cell-derived exosomes are taken up by TAMs. ↑: promoting effect; ↓: inhibitory effect; →: no effect; ?: unknown effect or unknown targets.
